# English and South African Dysentery

**Published:** 1901-10-05

**Authors:** 


### ENGLISH AND SOUTH AFRICAN DYSENTERY.

In a clinical lecture at Guy's Hospital, Dr. Washbourn,[1] after dealing with the cases in the wards, and referring to his experience of the disease in South Africa, spoke of the relationship between the South African and other forms of dysentery. He said that dysentery occurred to a not inconsiderable extent in English asylums, and that he had no doubt that this disease was identical with that which he had seen in South Africa. The fever and the other symptoms were the same, and the post-mortem appearances appeared to be identical, with the exceptions that acute cases of the English disease sometimes died before ulceration had occurred, a condition which he had not observed in South Africa. He had no doubt that the prevalence of dysentery in asylums was due to the same cause as its prevalence among troops on active service, viz., insanitary condition. Both in England and Africa the disease was infective, and Dr. Washbourn regarded the sporadic cases which had been described from time to time under the heading of simple colitis and ulcerative colitis as merely sporadic examples of the same disease as that which occurred in asylums. The form of dysentery which is described under the heading of tropical or amœbic dysentery was another affair and seemed to be distinct from the South African type. The course of amœbic dysentery was more prolonged and liver abscess was a common result. In the stools and in the pus from the abscess amœbæ were found, but this was certainly not the case with South African dysentery in which also liver abscess was decidedly rare. In regard to treatment absolute rest in bed was essential. The patient should not get up even to use the commode. The diet should consist of milk unless there were evidence that it was not being digested or that it disagreed, in which cases meat juice, whey, etc., should be substituted. The two specific modes of treatment were that by magnesium or sodium sulphate, and that by ipecacuanha. Sometimes when one had failed the other had succeeded. Magnesium sulphate was administered in drachm doses every hour until the motions assumed a fæcal character. If after 24 hours no effect was produced the treatment had better be left off. Ipecacuanha was administered thus:—After two or three hours' abstention from food a small dose of tincture of opium or chlorodyne was given, and half an hour later 30 grains of powdered ipecacuanha were administered. In three or four hours the dose was repeated. Dr. Washbourn said he had frequently made observations as to the value of the de-emetinised ipecacuanha, and he felt sure that it was not so good as the crude powder. Should diarrhœa continue after either form of treatment a mixture containing bismuth and morphia, or some other astringent, should be given, but care must be taken not to set up constipation. Where there was marked tenesmus morphia suppositories had a wonderful effect, and in some cases, where other treatment had failed, subcutaneous injections of morphia were very efficacious. Large rectal injections were sometimes of value in chronic cases, the injection consisting of simple warm water, or of some astringent, such, for example, as nitrate of silver half a grain to the ounce.

[1] Clinical Journal, Aug. 28.

